# Comparison of dendritic calcium transients in juvenile wild type and SOD1^G93A^ mouse lumbar motoneurons

**DOI:** 10.3389/fncel.2015.00139

**Published:** 2015-04-10

**Authors:** Katharina A. Quinlan, Jonathan B. Lamano, Julienne Samuels, C. J. Heckman

**Affiliations:** ^1^Department of Physiology, Feinberg School of Medicine, Northwestern UniversityChicago, IL, USA; ^2^Department of Physical Medicine and Rehabilitation, Feinberg School of Medicine, Northwestern UniversityChicago, IL, USA; ^3^Department of Physical Therapy and Human Movement Sciences, Feinberg School of Medicine, Northwestern UniversityChicago, IL, USA

**Keywords:** SOD1^G93A^ mice, motor neuron, spinal cord, calcium channels, multiphoton imaging

## Abstract

Previous studies of spinal motoneurons in the SOD1 mouse model of amyotrophic lateral sclerosis have shown alterations long before disease onset, including increased dendritic branching, increased persistent Na^+^ and Ca^2+^ currents, and impaired axonal transport. In this study dendritic Ca^2+^ entry was investigated using two photon excitation fluorescence microscopy and whole-cell patch-clamp of juvenile (P4-11) motoneurons. Neurons were filled with both Ca^2+^ Green-1 and Texas Red dextrans, and line scans performed throughout. Steps were taken to account for different sources of variability, including (1) dye filling and laser penetration, (2) dendritic anatomy, and (3) the time elapsed from the start of recording. First, Ca^2+^ Green-1 fluorescence was normalized by Texas Red; next, neurons were reconstructed so anatomy could be evaluated; finally, time was recorded. Customized software detected the largest Ca^2+^ transients (area under the curve) from each line scan and matched it with parameters above. Overall, larger dendritic diameter and shorter path distance from the soma were significant predictors of larger transients, while time was not significant up to 2 h (data thereafter was dropped). However, Ca^2+^ transients showed additional variability. Controlling for previous factors, significant variation was found between Ca^2+^ signals from different processes of the same neuron in 3/7 neurons. This could reflect differential expression of Ca^2+^ channels, local neuromodulation or other variations. Finally, Ca^2+^ transients in SOD1^G93A^ motoneurons were significantly smaller than in non-transgenic motoneurons. In conclusion, motoneuron processes show highly variable Ca^2+^ transients, but these transients are smaller overall in SOD1^G93A^ motoneurons.

## Introduction

The hallmark of amyotrophic lateral sclerosis (ALS) is the degeneration of both upper (corticospinal) and lower (spinal) motoneurons. Many disruptions have been found in both patients and animal models that could negatively affect motoneuron health, including altered protein degradation (Deng et al., 2011; Bendotti et al., 2012), neuroinflammation (Evans et al., 2013), and misregulated RNA processing (Polymenidou et al., 2012). Mutant superoxide dismutase-1 (SOD1) mouse models have yielded an abundance of information on properties of vulnerable motoneuron populations. Electrical and anatomical properties are altered from a very early age (Kuo et al., 2004, 2005; Bories et al., 2007; Amendola and Durand, 2008; van Zundert et al., 2008; Pambo-Pambo et al., 2009; Quinlan et al., 2011; Filipchuk and Durand, 2012; Martin et al., 2013; Leroy et al., 2014). Embryonically, mutant SOD1 spinal motoneurons have shorter dendritic branches and are hyperexcitable (Kuo et al., 2004, 2005; Martin et al., 2013), while postnatally spinal motoneurons show increased dendritic branching and their overall excitability is normalized, despite larger persistent inward Na^+^ and Ca^2+^ currents (NaPIC and CaPIC) (Amendola and Durand, 2008; Quinlan, 2011; Filipchuk and Durand, 2012). Whether the proliferated dendritic branches in mutant SOD1 motoneurons have similar levels of activity as dendrites of wild type motoneurons has not yet been examined.

The Ca^2+^ currents generating the CaPIC are most likely Cav1.3 (and potentially Cav1.2) channels. Though Cav1.3 channels failed to show higher expression levels in adult SOD1 motoneurons using immunohistochemistry (Shoenfeld et al., 2014), the larger amplitude current could reflect altered channel activation or kinetics rather than altered expression levels. Any potential changes in high-voltage activated Ca^2+^ currents (Cav2 channels) in SOD1 motoneurons remain relatively unstudied. Voltage-gated Ca^2+^ currents measured from wild type spinal motoneurons include P/Q (Cav2.1), N (Cav2.2), R (Cav2.3), L (Cav1.2 and 1.3), and T-type (Cav3) currents (Mynlieff and Beam, 1992; Carlin et al., 2000a). In brainstem hypoglossal motoneurons, previous studies have revealed complex microdomains of wildly differing internal Ca^2+^. Calcium that enters through voltage-gated Ca^2+^ channels is left unbound due to the lack of endogenous buffers, and is eventually taken up and released by the mitochondria and endoplasmic reticulum (Lips and Keller, 1999; Ladewig et al., 2003). The end result is variable internal Ca^2+^ arising from both internal stores and external Ca^2+^ entry in the soma and proximal dendrites (up to 80 μm) of hypoglossal motoneurons (Ladewig et al., 2003). In contrast to hypoglossal motoneurons, spinal motoneurons possess even more complex dendritic arborizations, but analysis of these microdomains remains unstudied.

Therefore, in this study there were two main goals: (1) To examine normal anatomical patterns of high-voltage activated Ca^2+^ transients elicited throughout the dendritic field of wild type motoneurons, (2) To compare the patterns of these transients in wild type motoneurons to those of SOD1^G93A^ motoneurons.

## Materials and methods

### Ethics statement

Experiments were performed in accordance with the United States National Institutes of Health Guide for Care and Use of Laboratory Animals. Approval of the Northwestern University's Animal Care and Use Committee was obtained for all experiments performed in this study. All efforts were made to minimize animal suffering and to reduce the number of animals used.

### Animals and surgery

For this study, 60 juvenile mouse pups were used between postnatal day (P) 4-11. Transgenic B6SJL mice overexpressing the human SOD1^G93A^ gene (strain 002726, Jackson Labs, Bar Harbor, ME, USA) were identified using standard PCR techniques (Rosen et al., 1993). Briefly, 20–25 mg of tissue was used for the DNA extraction with primers for amplification CAG TAA CTG AGA GTT TAC CCT TTG GT (forward) and CAC ACT AAT GCT CTG GGA AGA AAG A (reverse). Both transgenic SOD1^G93A^ and non-transgenic littermates were deeply anesthetized with isofluorane (Henry Schein Animal Health, Dublin, OH, USA), decapitated and eviscerated. The lumbar spinal cord was removed and embedded in 2.5% w/v agar (No. A-7002, Sigma-Aldrich, St. Louis, MO, USA). The agar block was then superglued with Loctite 401 (Henkel Corporation, Rocky Hill, CN, USA) to a stainless steel slicing bath and 350 μm transverse slices are made using the Leica 1000 vibratome (Leica Microsystems, Buffalo Grove, IL, USA) as described previously (Quinlan et al., 2011). During both spinal cord isolation and slicing, the spinal cord was immersed in 1–4°C high osmolarity dissecting solution containing (mM) sucrose 234.0, KCl 2.5, CaCl_2_ · 2H_2_O 0.1, MgSO_4_ · 7H_2_O 4.0, HEPES 15.0, glucose 11.0, Na_2_PO_4_ 1.0. The pH was adjusted to 7.35 when bubbled with 95% O_2_/5% CO_2_ using 1M KOH (Fluka Analytical, Sigma-Aldrich). After cutting, the slices were incubated for >1 h at 30°C in incubating solution containing (mM) NaCl 126.0, KCl 2.5, CaCl_2_ · 2H_2_O 2.0, MgCl_2_ · 6H_2_O 2.0, NaHCO_3_ 26.0, glucose 10.0, pH 7.4 when bubbled with 95% O_2_/5% CO_2_ (all reagents for solutions were purchased from Sigma-Aldrich).

### Electrophysiology

Whole cell patch clamp was performed on motoneurons from the lumbar segments using 2–4 MΩ glass electrodes pulled from glass capillary tubes (Item # TW150F-4, World Precision Instruments, Sarasota, FL, USA) with a Flaming-Brown P-97 (Sutter Instrument Company, Novato, CA, USA). Electrodes were positioned using a Sutter Instrument MP-285 motorized micromanipulator (Sutter Instrument Company). Whole-cell patch clamp measurements were performed at room temperature using the Multiclamp 700B amplifier (Molecular Devices, Burlingame, CA, USA) and Winfluor software (University of Strathclyde, Glasgow, Scotland). Briefly, slices were perfused with a modified Ringer's solution containing (in mM): 111 NaCl, 3.09 KCl, 25.0 NaHCO_3_, 1.10 KH_2_PO_4_, 1.26 MgSO_4_, 2.52 CaCl_2_, and 11.1 glucose. The solution was oxygenated with 95% O_2_ and 5% CO_2_ and the perfusion rate was 2.5–3.0 ml/min. Patch electrodes contained (in mM) 138 K-gluconate, 10 HEPES, 5 ATP-Mg, 0.3 GTP-Li and Texas Red dextran and Ca^2+^ Green-1 dextran (both 150 μM or both at 200 μM, 3000 MW, from Invitrogen, Life Technologies, Grand Island, NY, USA). The K*_d_* of Ca green-1 varied between lots, and ranged from 406 to 737 nM.

**Figure 1:**
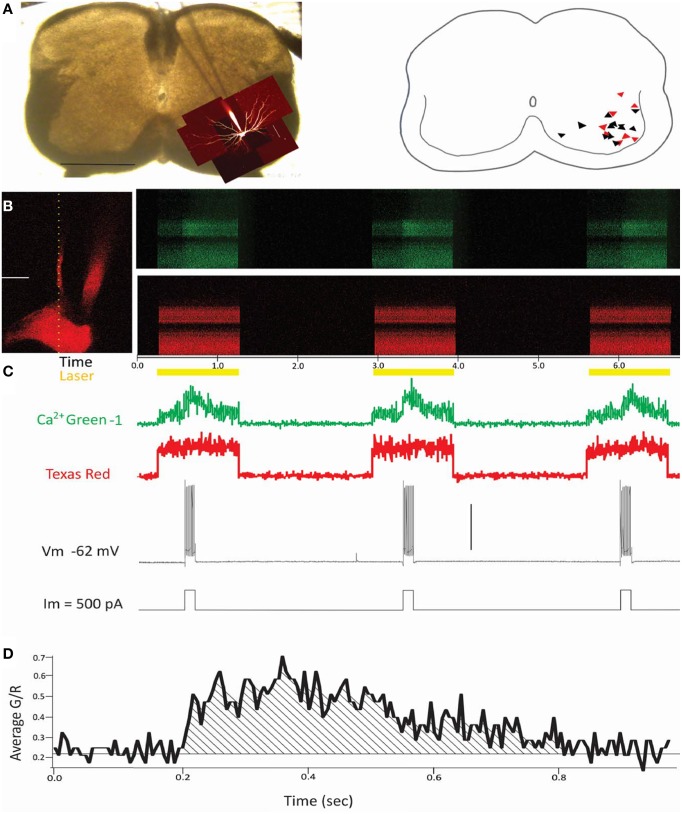
**Summary of experimental methods**. **(A)** Left panel: Photograph of a transverse spinal cord slice pictured with the patch electrode descending from the center top to the bottom right of the photo, where z-stacks of the filled cell are superimposed onto the cord. Horizontal scale bar = 0.5 mm. Right panel: Schematic showing placement of the putative motoneurons in black triangles (wild type, *n* = 13) and red triangles (SOD1, *n* = 8). **(B)** Left panel: Image of a patched neuron's soma and primary dendrite. The vertical dotted line indicates placement of the line scan. Scale bar = 20 um. Right panels: Green (top panel) and red (bottom panel) fluorescence is measured repeatedly over time (x axis) from the location of the line scan (dotted line from left panel of **B**). **(C)** Laser is turned on only during periods of simulation, as shown in yellow trace. Numeric values for green and red fluorescent intensity are below. The cell is stimulated with depolarizing pulses (200 ms/500 pA in this example) to fire action potentials (bottom two traces), during which the green channel shows a marked increase in fluorescence corresponding to the action potentials, while the red channel shows no change. Vertical scale bar = 50 mV. **(D)** The averaged signal for the three events, measured as Δ Green fluorescent intensity/Red fluorescent intensity over time. All values of Ca^2+^ transients throughout this study are calculated from the area under the curve of Green/Red fluorescent intensity, as shown in **(D)**.

During line scans, neurons were subjected to three depolarizing steps each at 50, 100, and 200 ms to evoke 1 action potential, 3–5 action potentials, and ~10 action potentials per step, respectively. Stimulation protocols were developed to assess activity of high voltage gated Ca^2+^ channels in the dendrites. Depolarizing the soma to threshold for a single spike elicited a fairly robust Ca^2+^ signal in the soma and proximal dendrites. However, it was not clear whether single spikes would be strong enough to spread electrotonically back as far as 300 um or more when more distal processes were examined. Conversely, limiting the duration of the stimuli was necessary to minimize the exposure time (and thus any damage) to the processes by the laser. With 50, 100, and 200 ms pulses, we avoided damaging dendrites with prolonged laser exposure yet produced a depolarization robust enough to measure even in distal dendrites. As shown in Figure 1, Ca^2+^ signals from the three events were averaged unless otherwise noted.

### Two-photon excitation fluorescence microscopy

An Olympus BX-51WIF microscope fitted with an Olympus 40x/0.8NA water-dipping objective lens was used for visualizing the patched neuron. Two photon excitation fluorescence microscopy (2PEF) was performed with a galvanometer-based Coherent Chameleon Ultra II laser (Coherent, Santa Clara, CA, USA). To choose an appropriate wavelength for excitation of both fluorophores, two photon excitation spectra were run for both Texas Red and Ca^2+^ green-1. Based on these spectra, the laser was tuned to 820 nm. The average laser power at this wavelength was about 2 W, though it is expected that some power was lost as the beam passed through the Bio-Rad Radiance modulators and the objective. Since emission of these fluorophores is well-separated, two Bio-Rad 2100 MPD photomultiplier tubes (Bio-Rad, Hercules, CA, USA): Green PMT (500–550 nm) and Red PMT (570–650 nm) were used to collect emission in the red and green channels. Unidirectional line scans were performed along the length of the dendrites with a dwell time of 10 μs/pixel. Precautions were taken to reduce noise in the images, including very low ambient light levels during experiments and a heavy curtain surrounding the rig. Fortunately, in the spinal cord of young mice at the ages used in these experiments, the auto-fluorescence of tissue is very low compared to the adult cord, probably due to incomplete myelination.

### Neuron selection

The ventrolateral motoneuron pools could be easily visualized in the slice preparation (as seen in Figure 1), and electrodes were positioned above this area. Putative motoneurons were targeted based on large soma diameter (>20 μM long axis). Only neurons with a resting potential <−50 mV, an action potential crossing 0 mV and a series resistance of <15 MΩ were used. Neurons were eliminated from analysis if series resistance or resting potential varied more than 10 MΩ or 10 mV, respectively, throughout the recording period. Cell properties are listed in Table 1.

**Table 1:** **Cell properties are listed for WT (*n* = 13) and SOD1 (*n* = 8) motoneurons**.

	**WT**			**SOD1**		
	**Mean**	**St Dev**	**Range**	**Mean**	**St Dev**	**Range**
R_IN_ (MΩ) *p* = 0.351	45	42	10 to 164	30	8	21 to 43
Cap (pF) *p* = 0.341	217	65	102 to 282	244	50	153 to 282
RMP (mV) *p* = 0.098	−61.1	4.8	−50.2 to 69.4	−64.7	4.3	−58.7 to 71.0
R_series_ (MΩ) *p* = 0.439	5.9	2.8	2.7 to 11.4	6.8	2.0	3.8 to 10.0

*Each parameter is listed with mean, standard deviation (St Dev), and range. Input resistance (R_IN_), capacitance (Cap), resting membrane potential (RMP), and series resistance (R_series_) are listed for all recorded cells. None of these parameters were significantly different between WT and SOD1 groups (p-values based on unpaired T-tests)*.

### Dendritic morphology

Neuronal tracing and three-dimensional reconstructions from z stacks were accomplished manually through Neurolucida reconstruction software (MBF Bioscience, Williston VT) and quantified with Neurolucida Explorer. Neuronal reconstructions, z stacks, and line scan reference images were utilized to determine the path distance from the soma to the line scanned processes, along with the mean diameter of the sampled segments.

### Action potential evoked Ca^+2^ entry

All signals throughout this manuscript are measured from the area under the curve of the averaged Ca^2+^ transient. In brief, custom software for MATLAB (Mathworks, Natick, MA, USA) was developed by our laboratory and used for precision measurement of Ca^2+^ signal along each line scan. For each line scan, a single position along the dendrite was retained, corresponding to the pixel that generated the largest Ca^2+^ signal for that line scan. The exact 0.2–0.3 μm region of the dendrite (after applying a moving average of range 2.6 μm) which gave rise to that peak signal was then assessed for (1) diameter and (2) path distance to the soma. The code is included as a Supplemental File.

### Regions of interest–dendrite vs. soma

Distinct regions of interest (ROI) along each line scan encompassing dendritic processes and somas were identified utilizing reference images and z stacks. Furthermore, an ROI devoid of processes was determined for each line scan to serve as a baseline intensity for normalizing observed Ca^2+^ Green and Texas Red intensities.

### Quantification of Ca^+2^ transient

Measurement of action potential evoked Ca^2+^ entry was achieved through analysis of the change in Ca^2+^ Green-1 fluorescent intensity through MATLAB. Ca^2+^ transients were analyzed following the onset of stimulus application until the end of imaging (approximately 1 s). For each scanned process, three evoked Ca^2+^ transients were quantified from each of three protocols. For all line scans, each pixel along the length of the scan was quantified temporally in terms of Ca^2+^ Green and Texas Red intensity normalized by the mean green and red fluorescent intensity within the baseline ROI, respectively. This method was pioneered by Bloodgood and Sabatini (2007). For each stimulus, the mean pre-stimulus baseline intensity of the Ca^2+^ Green fluorescence was subtracted from the Ca^2+^ Green intensity during the window from stimulus onset to imaging termination to determine the change in Ca^2+^ Green fluorescence (ΔG), an indicator of the change in internal Ca^2+^. ΔG was normalized by the pre-stimulus Texas Red fluorescence (R_0_) to obtain a ΔG/R_0_ curve. The area under the ΔG/R_0_ curve for each stimulus was then computed and the three values for each pixel of the line scan averaged together. As a result, a value for the area under ΔG/R_0_ was obtained for each pixel along the length of the line scan. For each line scan, the maximum value of area under ΔG/R_0_ within the dendritic or somal ROI was identified as a quantification of the Ca^2+^ transient, as shown in Figure 1D. From the pixel sampled as the maximum value of area under ΔG/R_0_, it was verified that the pixel was located within the process, then the path distance from that pixel to the soma was approximated in MATLAB based on neuronal reconstructions and line scan reference images.

### Within-cell branch analyses

For cells with scanned processes from at least three separate dendritic branches, a within-cell analyses of Ca^2+^ transients was performed using an arbitrarily assigned branch number as a variable in the analysis of covariance (ANCOVA) described below.

### Statistical analyses

All statistical analyses were performed using SPSS statistical software (IBM, Armonk, NY). Unless otherwise stated, Ca^2+^ transients were analyzed using ANCOVAs [modified ANOVAs tailored to use both categorical and quantitative variables (Fisher, 1971)]. This analysis was particularly suited to our dataset, since while testing the effect of one variable (i.e., path distance) on Ca^2+^ transients, the effects of covariates (i.e., stimulus duration) could be accounted for. Branch diameter and path distance were used individually in the analyses since there was significant interaction between the two variables (dendritic branches with the largest diameter were always closer to the soma). Unpaired *T*-tests were used to test significance of electrophysiological characteristics between WT and SOD groups. *T*-tests were used with *post-hoc* Bonferroni corrected multiple comparisons tests when testing baseline fluorescence.

## Results

### Action potentials initiated at soma elicited robust dendritic Ca^2+^ entry

In transverse spinal cord slices, eight SOD1^G93A^ and 13 wild type visually-identified motoneurons were recorded from the lumbar region as shown in Figure 1A. Large neurons were targeted in the motoneuron pools, and dyes from the electrode filled the patched neurons (Figure 1A, first and second panels). Electrophysiological characteristics of the motoneurons are included in Table 1. Texas red dextran (3000 MW) was used for its strong fluorescent signal, and Ca^2+^ transients were measured with the calcium indicator Ca^2+^ green-1 dextran (also 3000 MW). Stimulation protocols were run simultaneously with line scans as shown in Figures 1B,C. As the top (green) and bottom (red) panels in Figure 1B show, Texas red had a much stronger signal than Ca^2+^ green-1 at rest. But when action potentials were elicited, Ca^2+^ green showed a robust increase in fluorescence. Customized MATLAB software was used to measure the signal from 2.6 μm moving averages throughout the line scan and detect the pixel where the peak signal was centered. The anatomical characteristics of the dendrite at the location of the peak signal were used in later analysis. Three different protocols were applied to each neuron, each with three events: three depolarizing pulses of 50 ms (evoking one spike/stimulus), three pulses of 100 ms (evoking 3–5 spikes per stimulus), and three pulses of 200 ms (evoking about 10 spikes per stimulus). The three pulses of each protocol were averaged together (except where otherwise noted), and the area under the ΔGreen/Red curve (as shown in Figure 1D) was used to quantify the transient. This protocol was used in order to minimize the differences in laser penetration through different depths of the slice (by normalizing the fluorescence of the Ca^2+^ signal to the fluorescence of the inert Texas red), and to minimize changes in the peak and decay of the Ca^2+^ signal over time due to any potential increases in concentration of the Ca^2+^ sensitive dye over time (Helmchen et al., 1996).

### Baseline fluorescence

**Figure 2:**
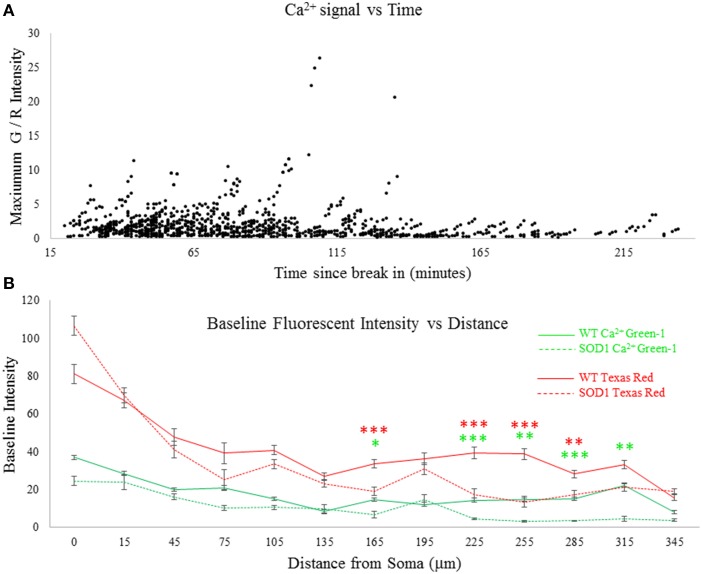
**Assessment of time and dye filling**. Patch electrodes with Ca^2+^ green-1 dextran and Texas red dextran in the internal solution filled motoneurons beginning at the time of break-in (marking the start of whole cell patch configuration). **(A)** Stimulation-evoked Ca^2+^ signal over time. There was a significant drop in the Ca^2+^ signal over time (*p* = 0.000), which was eliminated by excluding data past 120 min (*p* = 0.550). **(B)** Baseline fluorescence (without stimulation) was measured throughout all processes of all cells recorded. Fluorescent intensity in WT motoneurons is shown in solid lines and SOD1 in dotted lines. Significantly lower levels of both dyes are observed in SOD1 motoneurons in some dendritic compartments, suggesting impaired diffusion. Error bars = ±SEM, *T*-tests were used with *post-hoc* Bonferroni corrected multiple comparisons tests between WT and SOD1 for each fluorophore: *p < 0.05; **p < 0.01; ***p < 0.001.

After whole cell patch configuration was established, at least 20 min were allowed to pass for diffusion of the dyes. Since patch electrodes were large (2–4 MΩ resistance), diffusion took place rapidly and Texas red was clearly visible throughout the processes after this time. To further examine whether time may have had an effect on strength of the Ca^2+^ signal, ANCOVA was performed on the Ca^2+^ signal over time. It was found that after 120 min post-break in, there was a significant drop off in the Ca^2+^ signal, as can be observed in Figure 2A. This may indicate eventual run-down. Therefore, all data collected after 2 h was eliminated from further analysis. Data collected up to 120 min showed no significant effects of time, as shown in Table 2. The next concern was to ensure diffusion of dye was consistent between SOD1 and wild type motoneurons. Therefore, the baseline (at rest) fluorescent intensity of both Texas red and Ca^2+^-green-1 was measured throughout neuronal processes, as shown in Figure 2B. Differences in baseline [Ca^2+^]_internal_ could confound the interpretation of the Ca^2+^ green signal, but no such complication is exists for Texas red. Texas red showed a significantly lower fluorescent intensity in SOD1 motoneurons (dotted lines) than WT motoneurons (solids lines), starting at 165 μm from the soma. Though axonal transport has been shown to be altered in mutant SOD1 motoneurons (Zhang et al., 1997; Warita et al., 1999; Williamson and Cleveland, 1999; Kieran et al., 2005; De Vos et al., 2007; Bilsland et al., 2010), this phenomenon is not likely related. While much is still unknown about the intracellular mechanisms of dextran-conjugated dye spread, it does not rely on microtubule-dependent active transport (Fritzsch, 1993). Rather, passive diffusion of the dye from the soma to the distal dendrites could be impaired in high-protein-expressor SOD1^G93A^ neurons, or alternatively the dye could be more diffuse due to the larger size of SOD1 motoneurons (Amendola and Durand, 2008; Filipchuk and Durand, 2012). While this is in itself an interesting phenomenon, the problem of differential dye filling in measuring Ca^2+^ transients was overcome by normalizing the Ca^2+^ green-1 signal to the Texas red signal. In this way fluctuations in Ca^2+^ green-1 are normalized by the fluctuations of Texas red, since both are the same molecular weight and should be equally mobile in the cell. Thus, all signals are presented as the change in green fluorescent intensity/red fluorescent intensity (G/R Intensity).

**Table 2:** **Ca^2+^ transients are listed by category for WT (*n* = 13) and SOD1 (*n* = 8) motoneurons**.

	**WT Peak Ca^2+^ (ΔG/R Intensity)**				**SOD1 Peak Ca^2+^ (ΔG/R Intensity)**			
	**Mean**	**St Dev**	**Min**	**Max**	**Mean**	**St Dev**	**Min**	**Max**
**TIME FROM BREAK IN (min) *p* = 0.550**								
20–39	2.2	1.6	0.3	7.8	1.2	0.7	0.2	2.7
40–59	2.5	2.3	0.2	11.4	1.7	1.2	0.3	6.1
60–79	2.5	1.8	0.3	10.5	1.7	1.6	0.3	8.1
80–99	2.9	2.9	0.3	11.6	1.9	1.8	0.2	7.7
100–120	3.4	6.4	0.3	26.4	1.6	1.4	0.3	5.9
**PATH DISTANCE FROM SOMA (μm) *p* = 0.000**								
0–49	4.0	3.3	0.3	12.2	2.6	1.5	0.4	7.7
50–99	2.5	4.3	0.3	26.4	1.7	1.6	0.2	8.1
100–149	1.6	1.1	0.3	5.5	1.4	0.8	0.3	3.6
150–199	2.5	1.8	0.2	6.6	1.7	1.6	0.3	5.1
200–249	1.8	1.0	0.4	4.5	0.8	0.5	0.3	1.9
250–300	2.1	1.3	0.3	5.1	0.6	0.4	0.2	1.6
300 +	2.9	2.4	0.5	11.4	0.7	0.6	0.3	1.9
**DENDRITIC DIAMETER (μm) *p* = 0.000**								
0–1.4	2.3	1.5	0.3	5.7	1.5	1.4	0.2	5.1
1.5–2.9	2.3	2.7	0.2	26.4	1.5	1.3	0.3	8.1
3.0–4.4	3.8	3.4	0.4	12.2	2.4	1.0	1.2	4.6
4.5 +	4.5	3.6	1.1	8.8	2.6	1.9	1.4	7.7
**STIMULUS DURATION (ms) *p* = 0.004**								
50	2.3	2.6	0.2	22.4	1.5	1.2	0.2	7.1
100	2.6	2.8	0.3	24.9	1.7	1.4	0.3	8.1
200	2.9	3.2	0.3	26.4	1.8	1.6	0.3	7.7
**GENOTYPE *p* = 0.000**								
	2.6	2.9	0.2	26.4	1.7	1.4	0.2	8.1

*Each parameter is listed with mean, standard deviation (St Dev), minimum (Min), and maximum (Max). Ca^2+^ transients are listed in bins based on time, path distance from soma, dendritic diameter, and stimulus duration. Genotype values are also listed. Listed p-values are based on ANCOVA, and indicate the significance only of the listed factor (i.e., time) on Ca^2+^ transients (the p-value for genotype is listed only under the genotype heading)*.

### Progressive reduction in Ca^2+^ signal

**Figure 3:**
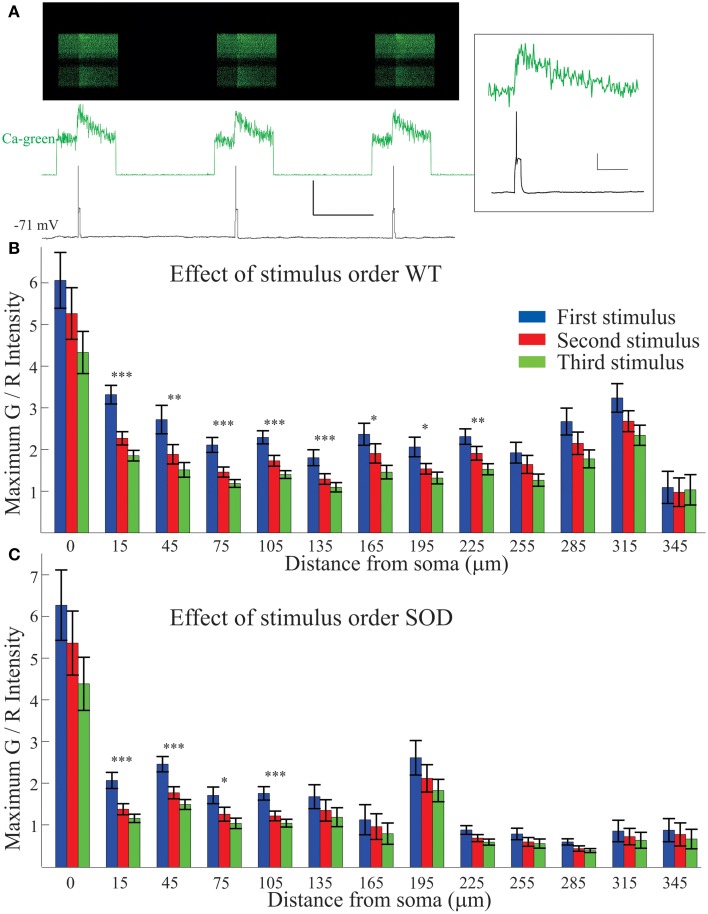
**Decrease in Ca^2+^ signal with repeated stimuli**. Each stimulus protocol (50, 10, and 200 ms depolarizing pulses) included three pulses of identical duration which were normally averaged together. The first stimulus pulse elicited the largest Ca^2+^ signal, as shown in a typical motoneuron in **(A)**, with 50 ms pulses. The Ca^2+^ green-1 fluorescent intensity (top two traces) shows a decreased Ca^2+^ signal in successive events though it returns to baseline after each event. The action potential (bottom trace) remains unchanged. Scale bar = 50 mV vertical, 1 s horizontal. Inset: High resolution zoom of the first stimulus. Scale bars = 20 mV vertical, 200 ms horizontal. **(B,C)** Each stimulus (first in blue, second in red and third in green) is averaged separately for WT **(B)** and SOD1 **(C)**. Over all the neurons recorded, significantly smaller Ca^2+^ transients were evoked with second and third events. Error bars = ±SEM, significance determined at each location with One Way ANOVA *p < 0.05; **p < 0.01; ***p < 0.001.

Each stimulus protocol was composed of 3 pulses of equal duration: three 50 ms pulses, (as shown in Figure 3A), three 100 ms pulses, and three 200 ms depolarizing pulses (shown in Figure 1C). Even the Ca^2+^ signals evoked with a single spike were quite robust (Figure 3A, top two panels and inset). As shown in Figure 3A, all Ca^2+^ transients returned to baseline after the stimulus ended. Any neurons that showed signs of bleaching or other photodamage were removed from the analysis. Also clearly visible from the Ca^2+^ green-1 trace (Figure 3A, middle panel), the first stimulus in the protocol elicited the largest response, and subsequent stimuli elicited smaller Ca^2+^ transients. Thus, Ca^2+^ signals from the 1st, 2nd, and 3rd pulses were plotted separately in Figures 3B,C. The graphs reveals two things: (1) an overall larger Ca^2+^ signal in the soma and proximal dendrites, and (2) a significantly larger Ca^2+^ transient for the first (blue) of the three stimuli at many points in the dendritic processes, even while action potential amplitudes remained consistent across stimuli (as in Figure 3A bottom panel). The drop off in Ca^2+^ signal could indicate Ca^2+^ channel inactivation, or activation of Ca^2+^ dependent K channels (SK channels). This phenomenon was most pronounced with the spike trains evoked by the 200 ms pulses, and least pronounced in the 50 ms single-spike stimulus protocol (data shown is average of all protocols).

### Predictors of calcium transient size

**Figure 4:**
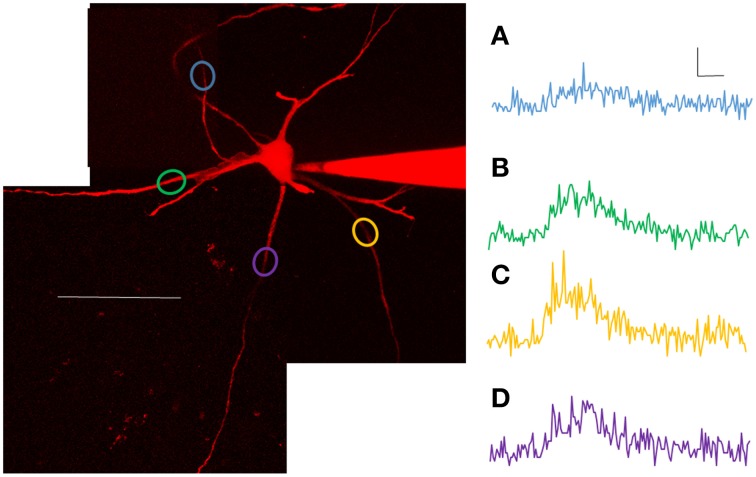
**Calcium transients varied significantly within motoneurons**. Based on ANCOVA, 3/7 motoneurons (2 of 4 WT and 1 of 3 SOD1) have branches with significantly different Ca^2+^ signals, over and above what is expected due to anatomical differences. Shown here are examples from one such motoneuron (P9 WT) pictured **on the left**. Scale bar = 100 μm. The variation in Ca^2+^ transients between processes can be appreciated from the plots in **(A–D)**, taken from points in the dendritic arbor at roughly equal path distances from the soma, elicited with 200 ms depolarizing pulses. **(A)** was taken at 120 min, **(B)** at 110 min, **(C)** at 56 min and **(D)** at 84 min. Scale bars for **(A–D)**: horizontal = 100 ms, vertical = 0.1 ΔG/R.

In order to capture anatomical characteristics, several z stacks were acquired after electrophysiological and Ca^2+^ measurements. To capture the full extent of the processes, multiple stacks were necessary. Generally, seven stacks were taken of neurons, though the exact number of stacks was dependent on the extent of the processes (range of 2 to 10 stacks). Stack sizes were 308 × 308 μm (512 × 512 pixels), and the depth ranged from 75 to 204 μm (step size = 1 μm). All of the morphological characteristics from the dendrites were compared to Ca^2+^ transients using ANCOVA. Ca^2+^ transients were found to correlate significantly with three factors: (1) number of spikes evoked/length of depolarizing current pulse, (2) distance from the soma, and (3) diameter of the dendrite at that location. More action potentials (from longer current steps), larger diameter dendrites and more proximal locations all contributed to larger Ca^2+^ signals (see Table 2 for complete breakdown of numbers). The simplest reason that all of those factors were significant is that they all affect the passive spread of current into the dendrites. However, in addition to those three factors, 3 of 7 motoneurons (1 of 3 SOD1^G93A^ and 2 of 4 wild type) showed significant variation from one dendrite to the next. In other words, even when the contributions of branch diameter, distance from the soma and stimulus length were accounted for (using ANCOVAs) 3/7 motoneurons had dendrites with significantly different profiles of Ca^2+^ entry. For example, compare the Ca^2+^ transients from the P9 wild type motoneuron shown in Figure 4. As shown, points A, B, C, and D have rather different Ca^2+^ transients. These examples are taken at equal distances from the soma, are averages of three 200 ms stimuli delivered while performing 2PEF at each location of the dendritic field. The dendritic diameter does vary somewhat between these processes, but it cannot account for the magnitude of difference between signals. Only motoneurons in which ≥3 branches had been sampled at overlapping distances were included in the analysis (*n* = 7). The processes with lowest and highest signals were not consistently first or last in the recording order, and data was eliminated from analysis whenever there was any sign of photodamage (including persistent increase in fluorescence after stimulation cessation; any blebbing of the process) or physiological weakening of the cell (changes in resting potential >10 mV or input resistance >10 MΩ). Rather, the different signals from a dendrite likely reflect Ca^2+^ microdomains as first suggested by Ladewig et al. (2003) in hypoglossal motoneurons. These microdomains could arise from altered current spread of the spike, altered presence of Ca^2+^ channels from dendrite to dendrite, local neuromodulation of the Ca^2+^ channels from one process to the next, or from different interactions of the external Ca^2+^ entry with Ca^2+^ from internal stores (also see Discussion).

### Decreased dendritic Ca^2+^ transients in SOD1^**G93A**^ motoneurons

**Figure 5:**
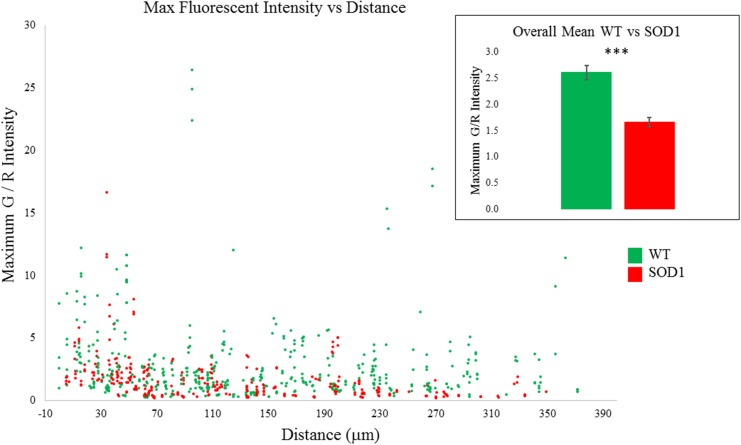
**SOD1 motoneurons had smaller Ca^2+^ transients than WT motoneurons**. WT (green) Ca^2+^ signals are shown along with SOD1 (red) along path distance from soma. Inset: average Ca^2+^ signal over all distances in WT and SOD1 (*p* = 0.000). Error bars = ±SEM, significance determined with ANCOVA.

Overall, eight SOD1^G93A^ and 13 wild type motoneurons were recorded. Comparing Ca^2+^ signals between these two groups, transients from SOD1^G93A^ motoneurons were significantly smaller (*p* = 0.000) as shown in Figure 5. This decrease appears to be more pronounced distally, likely reflecting a decrease in high voltage-activated Ca^2+^ channel activity in distal SOD1^G93A^ dendrites (see also Discussion). Table 2 includes data for each genotype by time, distance, dendritic diameter and stimulus duration. Potentially this could represent yet another early, homeostatic alteration of an intrinsic property in SOD1 motoneurons.

## Discussion

### Summary

Motoneurons showed significantly more robust Ca^2+^ transients with (1) longer depolarizing steps/increasing numbers of evoked action potentials, (2) larger dendritic diameters, and (3) shorter distances from the soma, as has been documented in other neuron types (Lips and Keller, 1999; von Lewinski and Keller, 2005; Day et al., 2008). Calcium transients with repeated stimuli showed tapering off of signal, perhaps evidence of Ca^2+^ channel inactivation or activation of SK channels. Remarkably, Ca^2+^ signals are highly variable from one dendrite to the next, with 3 of 7 motoneurons showing significantly different profiles of Ca^2+^ entry between processes in the same neuron. These results show the complexity of local microdomains in motoneuron processes. Lastly, the Ca^2+^ transients were significantly smaller in SOD1^G93A^ motoneurons. This could be due to lower Ca^2+^ channel expression/activation, or both.

### Previous work on motoneuron Ca^2+^ currents/channels

Spinal motoneurons display a wide array of Ca^2+^ currents. L-type Cav 1.3 channels contribute to the CaPIC, one of the currents which sets overall neuronal excitability. The PICs are also of particular interest since they are elevated in SOD1 motoneurons at this age. Cav 1.3 channels are found on both proximal and distal dendrites with a patchy distribution, probably at the sites of synaptic contact (Westenbroek et al., 1998; Carlin et al., 2000b; Simon et al., 2003; Zhang et al., 2008a). The uneven channel distribution and modeling suggest the presence of “hotspots” along the dendritic field where Cav1.3 channels are clustered (Grande et al., 2007). Though our data does appear to show hotspots in the dendritic field, our stimulation protocol is unlikely to activate Cav1.3 type Ca^2+^ channels. If we were activating CaPICs, we would probably not see a return to baseline in either the membrane voltage or the Ca^2+^ signal. Instead, we do see a repolarization of the membrane and decreasing, rather than increasing Ca^2+^ transients with each stimulus. The other L-type Ca^2+^ channels, the Cav1.2 channels, are quite likely contributing to Ca^2+^ signals in this study. They show intense labeling in motoneuron somata and proximal dendrites (Westenbroek et al., 1998; Simon et al., 2003), and along with the Cav1.3 channels have an increasing presence during postnatal maturation until P18 (Jiang et al., 1999). Due to the strong Ca^2+^ signals we observed from these areas and the mean age of the neurons recorded (P8), these channels probably are contributing to the Ca^2+^ signals here, along with the other high-voltage-activated Ca^2+^ channels Cav2.1 (P/Q) and Cav2.2 (N) type channels. Though classically the Cav2.1 channels mediate vesicle release at axon terminals, they have also been shown to contribute substantially to whole cell Ca^2+^ currents measured at motoneuron somata (Umemiya and Berger, 1995; Carlin et al., 2000a). Cav2.2 have been found on motoneuron somata and proximal dendrites based on immunohistochemistry, as have Cav2.3 (R) type channels (Westenbroek et al., 1998), and could also be contributing to our Ca^2+^ signals.

### Attenuation of Ca^2+^ transients

As shown in Figure 3, successive stimuli evoked smaller Ca^2+^ transients. Inactivation of high-voltage activated Ca^2+^ channels has been previously described (Catterall et al., 2013). However, another possibility is that successive activation of Ca^2+^ channels during spiking is sufficient to open SK (Ca^2+^-activated K^+^) channels. SK channels are present on motoneurons (Safronov and Vogel, 1998), and their activation depends both on voltage and the presence of Ca^2+^ (Li and Bennett, 2007; Abbinanti and Harris-Warrick, 2012). As Ca^2+^ influx from the first pulse activates SK channels, the increased K^+^ conductance would allow less voltage change in subsequent pulses. Dampening the dendritic depolarization would in turn dampen the Ca^2+^ signal.

### Dendrites are highly variable

Landmark studies by Jaffe et al. (1992, 1994) showed Ca^2+^ transients throughout the dendrites in hippocampal pyramidal neurons depend on dendritic invasion of the Na^+^ spike. The strength of the dendritic Ca^2+^ signal was shown to attenuate steeply in distal dendritic compartments that lack voltage dependent Na^+^ channels, the degree of decline depending on intracellular resistance of the cell (Jaffe et al., 1992, 1994). In fact, spatial gradients in Ca^2+^ signals could be achieved in models without any changes in Ca^2+^ channel density throughout the processes (Jaffe et al., 1994). Instead, differing current spread in each compartment could explain the difference between processes. In the case of the spinal motoneurons, the anatomical contributions to varying Ca^2+^ signals are compounded by varied Ca^2+^ channel density as well. Cav1.3, 2.1, and 2.2 channels have been described as having a patchy distribution, or regions of higher density, along the length of motoneuron dendrites (Westenbroek et al., 2005). Moreover, hypoglossal motoneurons show local Ca^2+^ microdomains arising from the concert of activity in voltage-gated Ca^2+^ channels, located in varying proximity to Ca^2+^ sequestering organelles which are themselves non-uniformly distributed in the cell (Lips and Keller, 1999; Ladewig et al., 2003). Hypoglossal motoneurons and spinal motoneurons appear to share these Ca^2+^ microdomains, with spinal motoneurons showing significantly different Ca^2+^ signals from one dendrite to the next in 3/7 motoneurons analyzed. Additionally, our data supports the existence of “hotspots” in high voltage gated Ca^2+^ channels. Hotspots have been previously described in low voltage gated Ca^2+^ channels (Bui et al., 2006; Elbasiouny et al., 2006; Grande et al., 2007; Carlin et al., 2009). Another factor that could also be contributing to the complexity of the Ca^2+^ signals is local neuromodulation of Ca^2+^ channels. Serotonin (5HT2R), metabotropic glutamate, and adenosine A1 receptor activation can all suppress Cav2 channel activity (Mynlieff and Beam, 1994; Bayliss et al., 1995; Del Negro and Chandler, 1998; Ladewig et al., 2004). Serotonin has even been shown to differentially modulate Ca^2+^ signals: Ca^2+^ transients at certain dendritic sites are strongly depressed by serotonin while other dendritic sites on the same neuron are unaffected (Diaz-Rios et al., 2007).

### Ca^2+^ entry and synaptic inputs to SOD1 motoneurons

Motoneurons that are vulnerable to degeneration in ALS (and mutant SOD1) pathology are the largest, fast fatigable motoneurons which lack Ca^2+^ buffering proteins (Lips and Keller, 1998, 1999; Palecek et al., 1999b; Pun et al., 2006; Hegedus et al., 2007), in fact they have Ca^2+^ buffering capacities that are 5 to 6 times lower than found in disease-resistant motoneurons (Lips and Keller, 1998; Palecek et al., 1999a). The mitochondria are largely responsible for uptake of unbound internal Ca^2+^. Near disease end stage in hypoglossal motoneurons, Ca^2+^ handling is completely remodeled such that there is decreased dependence on the mitochondria for Ca^2+^ uptake and increased plasma membrane extrusion (Fuchs et al., 2013). Altered Ca^2+^ sequestering and an exaggerated response of the electrical gradient of the inner membrane to stimulation-induced Ca^2+^ influx has also been demonstrated (Damiano et al., 2006; Bilsland et al., 2008; Jaiswal et al., 2009; Nguyen et al., 2009; Li et al., 2010). These studies may indicate a lifelong battle for Ca^2+^ homeostasis in motoneurons. In the juvenile age studied here, current results suggest that high voltage-activated Ca^2+^ channels may be less active (or less prevalent) in SOD1 motoneurons, while in contrast, the Cav1.3 channels responsible for the PIC appear to be more active (Quinlan et al., 2011). Interestingly, cultured wild type spinal motoneurons exposed to SOD1^G93A^ astrocyte conditioned media showed somewhat similar results (Fritz et al., 2013). These motoneurons developed electrical similarities to SOD1^G93A^ motoneurons (including larger PICs). They also showed reduced amplitude dendritic Ca^2+^ transients evoked by spontaneous synaptic events (though the frequency of these spontaneous events increased). Since this study used the membrane permeable Ca^2+^ indicator Fura-2 AM, dye diffusion would not be a factor here (Fritz et al., 2013). The contribution of the synaptic machinery to the activation of dendritic Ca^2+^ channels should not be overlooked. Herron and Miles (2012) describe enlarged C-boutons on motoneurons of male, but not female SOD1 mice. Bories et al. (2007) reported smaller amplitude depolarizations in membrane voltage of spinal motoneurons evoked with dorsal root stimulation, while in hypoglossal motoneurons, van Zundert et al. (2008) observed an increased frequency of spontaneous AMPAergic and GABAergic events with no change in current amplitude. Further study is critically needed to determine the exact contributions of motoneuron Ca^2+^ channels and altered synaptic inputs in motoneuron vulnerability. This work is increasingly possible since great strides have been made in identifying neurons which synapse directly onto spinal motoneurons (Butt and Kiehn, 2003; Miles et al., 2007; Quinlan and Kiehn, 2007; Crone et al., 2008; Zhang et al., 2008b; Kasumacic et al., 2010; Bui et al., 2013; Talpalar et al., 2013). Indeed, a recent study indicates alterations in synaptic inputs and the cell signaling pathway mTOR could be a very promising target for treatment of ALS (Saxena et al., 2013).

### Dendrites of SOD1 motoneurons

Our results show for the first time that high expressor SOD1 motoneurons may have an impedance to dye filling. It would be tempting to assume dendritic transport is altered similarly to axonal transport (Zhang et al., 1997; Warita et al., 1999; Williamson and Cleveland, 1999; Kieran et al., 2005; De Vos et al., 2007; Bilsland et al., 2010). However, transport of dextrans occurs independent of microtubule-dependent active transport (Fritzsch, 1993). Since protein expression levels in the SOD1 model used here are orders of magnitude higher than in non-transgenic mice, perhaps it has reached a point that impedes diffusion of dye. Alternatively, the dendritic arborization is expanded in SOD1 spinal motoneurons (Amendola and Durand, 2008; Filipchuk and Durand, 2012), so with potentially more dendrites to fill, it could be that the dye was just less effective at filling an expanded area. Despite the limitations in dye filling, many steps were undertaken to normalize Ca^2+^ signals between regions that were differently filled with dye, or experienced differences in laser penetration (processes that were deeper in the slice than others). Specifically, use of 3000 MW Texas red to normalize transients detected by 3000 MW Ca^2+^ green-1 can control for these variables.

## Conclusions

Intracellular Ca^2+^ has broad impact on neurons, affecting the immediate electrophysiology, the short term cell signaling pathways, and in the long term, neurodegenerative pathways. In the present study we show that dendritic Ca^2+^ entry is highly variable even in wild type motoneurons. This likely allows for the fine tuning of synaptic inputs and intrinsic cell currents through neuromodulatory processes and variable expression of dendritic Ca^2+^ channels. In the SOD1^G93A^ motoneurons, the complexity of Ca^2+^ entry is certainly not diminished. Dendritic Ca^2+^ entry can be significantly different from one branch to the next, and the overall Ca^2+^ signal in SOD1 motoneurons is smaller.

## Author contributions

KQ designed, performed and analyzed the experiments, performed statistical analysis, interpreted the data and wrote the manuscript. JL designed the customized software to match transients to their anatomical parameters, performed the data analysis and statistical analysis. JS assisted with data analysis. CH developed the initial conception of these experiments, interpreted the data, and contributed to the manuscript.

### Conflict of interest statement

The authors declare that the research was conducted in the absence of any commercial or financial relationships that could be construed as a potential conflict of interest.
